# How does academic self-efficacy influence learning anxiety and academic burnout in Chinese characters learning among international students in China?

**DOI:** 10.3389/fpsyg.2025.1555063

**Published:** 2025-07-01

**Authors:** Yang Lin

**Affiliations:** Faculty of Humanities and Foreign Languages, Xi’an University of Technology, Xi’an, China

**Keywords:** academic self-efficacy, learning anxiety, academic burnout, Chinese character learning, international students in China

## Abstract

**Background:**

The interplay between learning self-efficacy, anxiety, and burnout has been extensively documented in English as a Second Language (ESL) education. However, Chinese character learning—a uniquely complex task involving visual–spatial processing, stroke order mastery, and radical decomposition—presents distinct cognitive and affective challenges. The dynamics of self-efficacy, anxiety, and burnout in Chinese character acquisition remain under-explored, creating a critical gap in understanding how these constructs operate in non-alphabetic language contexts.

**Objective:**

The purpose of this study was to investigate how learning anxiety (LA) affects academic burnout (AB) and explores the role of academic self-efficacy (ASE) in the relationship between the two.

**Methods:**

A study of 537 international students (50.4% males, mean age = 20.96 years, SD = 1.36) was conducted using the Academic Self-Efficacy Scale (ASES), Foreign Language Learning Anxiety Scale (FLLAS), Academic Burnout Scale (ABS).

**Results:**

① LA was significantly and positively correlated with AB, and significantly and negatively correlated with ASE. ② ASE mediated the relationship between LA and AB. ③ Grade level, sleep quality, and parental education level have a significant effect on ASE, LA and AB; and ④ Extroversion has a significant effect on ASE, but not on LA and AB.

**Conclusion:**

The chain mediation model validated by this study provides valuable insights into the effects of international students’ learning anxiety (LA) on academic burnout (AB) in China, alongside practical implications for preventing and intervening in LA and AB among other current students.

## 1 Introduction

In foreign language learning, learners’ individual factors have an important impact on learning effectiveness (Li, 2015), which include psychological attributes such as motivation, learning strategies, learning cognition, self-efficacy, learning anxiety and burnout (Dörnyei, 2005). Among them, academic self-efficacy (ASE), learning anxiety (LA) and academic burnout (AB) have received extensive attention from scholars because of their far-reaching effects on learning effectiveness (May et al., 2015; Lyndon et al., 2017; Li et al., 2021).

Recent SLA research has increasingly embraced positive psychology perspectives (Dewaele et al., 2019), emphasizing the dynamic interplay between affective factors and language learning outcomes (Dewaele and MacIntyre, 2014; Krashen, 1985; MacIntyre and Gregersen, 2012), with control-value theory (Pekrun, 2006) highlighting how learning emotions shape both processes and performance. In Chinese character learning specifically, LA triggers particularly strong negative emotional responses due to three cultural-linguistic factors: (1) cognitive-cultural dissonance from the logographic system’s demands on alphabetic-L1 learners (Wang et al., 2005; Shen, 2013; Liu and Hsieh, 2016), (2) Confucian-heritage educational values emphasizing perfectionism that may conflict with learners’ native cultural scripts (Tweed and Lehman, 2002; Tran et al., 2012), and (3) pedagogical mismatches between traditional character-teaching methods and learners’ culturally-embedded expectations (Zhang and Zhu, 2025; Xu et al., 2021), with research showing Japanese kanji-experienced learners demonstrate 32% lower anxiety (Lu, 2022), collectively suggesting Chinese character learning constitutes both a cognitive and cultural-adaptive process where affective responses are mediated by native scripts, educational socialization, and cultural values (Dewaele and Li, 2021).

Currently, studies have shown that ASE is significantly negatively correlated with LA and AB (Bandura, 1997; Mills et al., 2007; Richardson et al., 2012), but existing studies have mostly focused on one dimension (Lee and Jeon, 2015), and there is a scarcity of research that comprehensively explores the interrelationships among these three dimensions (Charkhabi et al., 2013). In order to fill this gap, this study adopts quantitative analysis to analyse the mediating role of ASE between LA and AB, and to provide new perspectives and empirical evidence for the study of psychological factors of Chinese character learners.

## 2 Literature review

### 2.1 Conservation-of-resources theory

Developed by Hobfoll (1989), the conservation-of-resources (COR) theory posits that individuals are motivated to preserve, protect, and accumulate resources to mitigate stress and enhance well-being. The theory emphasizes two core principles: (1) resource loss threat (the anticipation of resource depletion) is a primary driver of stress, and (2) individuals with abundant resources are better equipped to withstand stressors through resource investment strategies (Hobfoll et al., 2018). In educational contexts, COR theory has been applied to explain how students’ personal resources (e.g., self-efficacy, academic motivation) influence their ability to manage academic stressors and avoid burnout (Schaufeli and Taris, 2005).

Within the COR framework, academic self-efficacy serves as a critical enduring personal resource that enables students to resist resource loss threats (e.g., buffering perceived challenges in Chinese character learning), facilitate resource investment (e.g., engaging in proactive behaviors such as deliberate practice or seeking social resources like teacher feedback), and mitigate resource spirals (e.g., countering the downward spiral of low self-efficacy leading to task avoidance and skill deficits) (Hobfoll et al., 2018). For international students, Chinese character learning presents unique resource-demanding stressors including high cognitive load from the arbitrary mapping of glyphs, pronunciation, and meaning that risks resource depletion (Shen, 2005), cultural and linguistic distance that exacerbates feelings of incompetence and anxiety for non-Chinese (alphabetic) language users (Chai and Bao, 2023), and performance pressure to achieve fluency in a non-logographic script that amplifies perceived resource loss threats, especially for those with low self-efficacy (Bassetti et al., 2020). This study bridges this gap by investigating how ASE, as a core resource, interacts with Chinese-specific stressors to influence LA and AB, thereby offering empirical validation of COR theory in this specialized academic field.

### 2.2 LA and AB

Among factors influencing AB, LA stands as a critical predictor (Lepp et al., 2014). LA, characterized by fear and apprehension in academic settings (Horwitz et al., 1986), is exacerbated in foreign language learning due to linguistic and cultural complexities (Spielberger, 1972). For learners of Chinese, the ideographic writing system imposes unique cognitive demands on phonological, semantic, and textual processing (Saito et al., 1999; Taylor and Taylor, 1995; Wang et al., 2005), while cultural connotations embedded in characters amplify comprehension anxiety for those unfamiliar with Chinese culture (Liu, 2010; Li, 2019; Liu, 2023). Prolonged LA often escalates into AB—a state of emotional and mental exhaustion marked by disillusionment and reduced efficacy (Freudenberger, 1974; Wu, 2020; Schaufeli et al., 2002). In Chinese language acquisition, AB is further compounded by the cognitive load of mastering approximately 3,000 characters (Sun, 2025; Luo et al., 2025), academic pressures (Fan and Hu, 2010), and cultural adaptation challenges (Mokhtar and Abidin, 2021), alongside factors like perfectionism (Zhang et al., 2007) and social isolation (Mulyadi et al., 2016). Early interventions, such as adaptive strategies and socio-emotional support, are vital to mitigating these issues (Kan et al., 2025). This study hypothesizes that LA significantly predicts AB (H1), underscoring the urgency of addressing their interplay in cross-cultural language education.

### 2.3 ASE as a mediator

Academic Self-Efficacy (ASE) refers to learners’ confidence in their ability to organize and execute actions required to achieve academic goals (Bandura, 1977). In this study, ASE specifically measures learners’ perceived competence in mastering Chinese character learning tasks. In the context of learning, self-efficacy plays a crucial role in determining students’ motivation, effort and persistence (Bandura, 1986; Martin and Rimm-Kaufman, 2015). From a social and positive psychology perspective, ASE influences learners’ choice and use of language learning strategies, and those with high self-efficacy are more likely to adopt active, meta-cognitive strategies, and they are closely linked to motivation (Zimmerman, 2000). Research has indicated that ASE influences academic motivation (Zimmerman and Schunk, 2011), academic performance (Caprara et al., 2011), academic achievement (Caprara et al., 2011), academic stress (Mulyadi et al., 2016), academic hardiness (Cheng et al., 2019), engagement (Schunk and DiBenedetto, 2020) and behavior (Britner and Pajares, 2006). Current research has focused more on teachers’ self-efficacy (Dewaele and Leung, 2022; Wang and Pan, 2023) and less on students’ self-efficacy (Han and Wang, 2021; Orakcı et al., 2023; Yang, 2021). Therefore, there is a need to further explore ASE and its relationship with other academic emotions, as well as the impact of these factors on Chinese character learning. The current study hypothesizes that academic self-efficacy (ASE) serves as a mediator in the relationship between learning anxiety (LA) and academic burnout (AB) (H4).

### 2.4 Control-value theory and the present study

**Figure 1:**
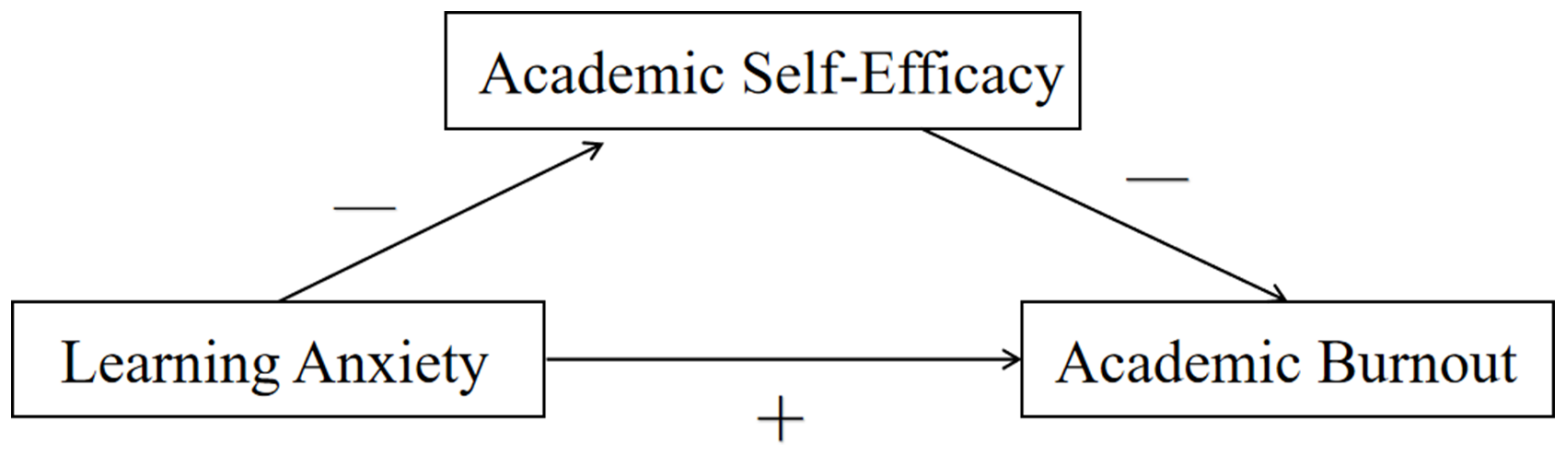
Graphical overview of the hypothesized effects.

Control-Value Theory (CVT) emphasizes the dynamic interaction between a learner’s emotions, motivation, and academic achievement, with a focus on how perceptions of control and value influence these factors. The relationship between ASE, LA and AB is a dynamic and interconnected process, heavily influenced by the perceptions of control and value that underlie Control-Value Theory. Current research supports the claim that teachers’ self-efficacy plays a crucial role in predicting their engagement in teaching (Wang and Pan, 2023). Given the assumption that learners’ ASE can influence LA and AB, and considering the significant role of ASE in predicting academic engagement in the educational context, a hypothetical model (Figure 1) is proposed to examine how these constructs interact.

The model will be based on the following research hypotheses:

*H1*: Does learners’ LA directly impact AB?

*H2*: Does learners’ LA directly impact ASE?

*H2a*: Does language anxiety dimension directly impact ASE?

*H2b*: Does classroom anxiety dimension directly impact ASE?

*H2c*: Does test anxiety dimension directly impact ASE?

*H2d*: Does negative evaluation anxiety dimension directly impact ASE?

*H3*: Does learners’ ASE directly impact AB?

*H3a*: Does learners’ ASE directly impact feeling depressed dimension?

*H3b*: Does learners’ ASE directly impact misconduct dimension?

*H3c*: Does learners’ ASE directly impact low sense of accomplishment dimension?

*H4*: Does learners’ LA indirectly impact AB via the mediation of ASE?

*H5*: Do graduate students report significantly higher levels of LA and AB, but lower ASE, compared to undergraduate students?

*H6*: Does grade level, sleep quality, and parental education significantly predict international students’ ASE, LA, and AB in Chinese character acquisition?

## 3 Research methodology

### 3.1 Participants

Using the multistage sampling method, all international students from 6 universities in Shaanxi Province in China, a total of 550 students, were selected to complete the questionnaire survey, and a total of 537 valid questionnaires were returned, with a valid return rate of 97.6%. Among them, 271 (50.4%) were male and 266 (49.5%) were female; 296 were Bachelor’s degree students and 241 were Master’s degree students aged 18 to 25 (M_age_ = 20.96, SD = 1.36). The distribution by academic year was as follows: 91 first-year undergraduates, 74 s-year undergraduates, 64 third-year undergraduates, 67 fourth-year undergraduates, 87 first-year postgraduates, 81 s-year postgraduates, and 73 third-year postgraduates. Participants represented diverse linguistic backgrounds (42% Indo-European, 33% Afro-Asiatic, 15% Sino-Tibetan, 10% other) and Chinese proficiency levels (HSK5-6: 51.2%, HSK3-4: 27.7%, HSK1-2: 21.0%). Socioeconomic indicators revealed that 61% of participants had at least one parent holding a bachelor’s degree or higher, 54% reported family incomes exceeding their home countries’ national medians, and 39% had accessed paid private language tutoring during their Chinese studies. These international students came from different contries and majors, and it can be confirmed that the sample drawn is highly representative of the international students population.

### 3.2 Instruments

#### 3.2.1 Academic Self-Efficacy Scale

The *Academic Self-Efficacy Scale (ASES)*, developed by Schwarzer and Aristi (1997) and revised in Chinese by Zhang and Schwarzer (1995) was employed to measure students’ perceived competence in achieving academic goals. This instrument was selected because it is a well-validated tool widely used in educational psychology, particularly in cross-cultural contexts (Pajares, 1996; Bandura, 1997; Klassen and Usher, 2010; Honicke and Broadbent, 2016). Its uni dimensional structure (14 items) assesses overarching academic confidence, avoiding domain-specific limitations. Sample questions such as “I can find effective ways to learn Chinese characters,” “I am satisfied with my progress in learning Chinese characters so far” and “I am confident that I will do well in exams or writing tasks” are scored on a 5-point scale. The higher the total score is, the stronger the academic self-efficacy is. The total Cronbach’s alpha coefficient of the scale was 0.945. Confirmatory factor analysis (CFA) was conducted on the 14 items; the average variance extracted (AVE) of each second-order factor is 0.551 (> 0.5), and the composite reliability (CR) is 0.945 (> 0.7). The results of the model showed that CMIN/DF = 1.057 (< 3), RMSEA = 0.010 (< 0.08), and RMR = 0.022 (< 0.05); the GFI, CFI, NFI, and IFI values were 0.979, 0.999, 0.982, and 0.999, respectively, which were all greater than 0.9, indicating that the results of CFA had good fitting indicators.

#### 3.2.2 Learning Anxiety Scale

The *Foreign Language Learning Anxiety Scale* (FLLAS; Horwitz et al., 1986) was employed to assess students’ anxiety levels in language learning. This instrument was selected because it is the most widely validated measure of language-specific anxiety (Dewaele and MacIntyre, 2014) and has demonstrated cross-cultural reliability in Asian educational contexts (Liu, 2006). The scale involves four main dimensions and 20 questions, of which vigor corresponds to language anxiety (items LA1—LA4, e.g., “I am afraid of learning Chinese characters.”), foreign language classroom teaching anxiety (items CA1—CA6, eg., “I do not like Chinese characters class.”), test anxiety (items TA1—TA4, e.g., “My palms get sweaty and my heart beats faster during the Chinese characters test.”) and negative evaluation anxiety (items NEA1—NEA5,e.g., “I will be afraid that my classmates will make fun of my Chinese characters level.”) and is a Likert scale with five points, in which 1 symbolizes strongly disagree and 5 indicates strongly agree. The total Cronbach’s alpha coefficient of the scale was 0.927. Confirmatory factor analysis (CFA) was conducted on 20 items; the average variance extracted (AVE) is 0.550, and the composite reliability (CR) is 0.857. The results of the model showed as follows: CMIN/DF = 1.164 (< 3), RMSEA = 0.017 (< 0.08), and RMR = 0.035 (< 0.05), and the values of GFI, CFI, NFI, and IFI are 0.966, 0.994, 0.960, and 0.994, respectively, which are greater than 0.9, indicating that the results of CFA had good fitting indicators.

#### 3.2.3 Academic Burnout Scale

The revised scale was adopted based on *Academic Burnout Scale* (ABS) with three dimensions of feeling depressed (FD), misconduct (MI) and low sense of accomplishment (LSA), a total of 12 items (Zhao, 2019). This instrument was selected because it has been specifically validated for Chinese university populations and captures three clinically significant dimensions of academic burnout. Sample questions such as feeling depressed “When I wake up in the morning, I have little energy at the thought of having to attend a class on reading and writing Chinese characters,” and misconduct “In the Chinese characters reading and writing class, my mind wanders and I do not know what to think about,” and low sense of accomplishment “I feel exhausted after one lesson.” The scale was scored on a 5-point Likert scale, with all positive scores, one score for “Not at all” and five scores for “Fully,” and the higher the total score, the more serious the degree of academic burnout. The total Cronbach’s alpha coefficient of the scale is 0.869. Confirmatory factor analysis (CFA) was conducted on 12 items; the average variance extracted (AVE) of each second-order factor is 0.570, and the composite reliability (CR) is 0.841, indicating thata the aggregation validity is high. The results of the model showed that CMIN/DF = 1.083 (< 3), RMSEA = 0.012 (< 0.08), RMR = 0.031 (< 0.05), GFI = 0.984, CFI = 0.998, NFI = 0.980, IFI = 0.998, indicating that the results of CFA had good fitting indicators.

## 4 Results

### 4.1 Correlation between LA, AB and ASE

Table 1 presents in detail the means, standard deviation and correlation coefficients of the main variables. Table 1 shows that all variables are significantly correlated with each other (*p* < 0.01), with LA having a significant positive correlation with AB (r = 0.606, *p* < 0.01); and ASE having a significant negative correlation with AB (r = −0.525, *p* < 0.01) and LA (r = −0.471, *p* < 0.01), which verifies H1, H2, H3 hold true.

**Table 1:** Means, standard deviations and correlation coefficients of the variables.

Variable	M	SD	1	2	3
LA	3.20	0.631	1		
ASE	2.82	0.880	−0.471**	1	
AB	3.19	0.710	0.606**	−0.525**	1

**p < 0.01.

### 4.2 SEM analysis

**Figure 2:**
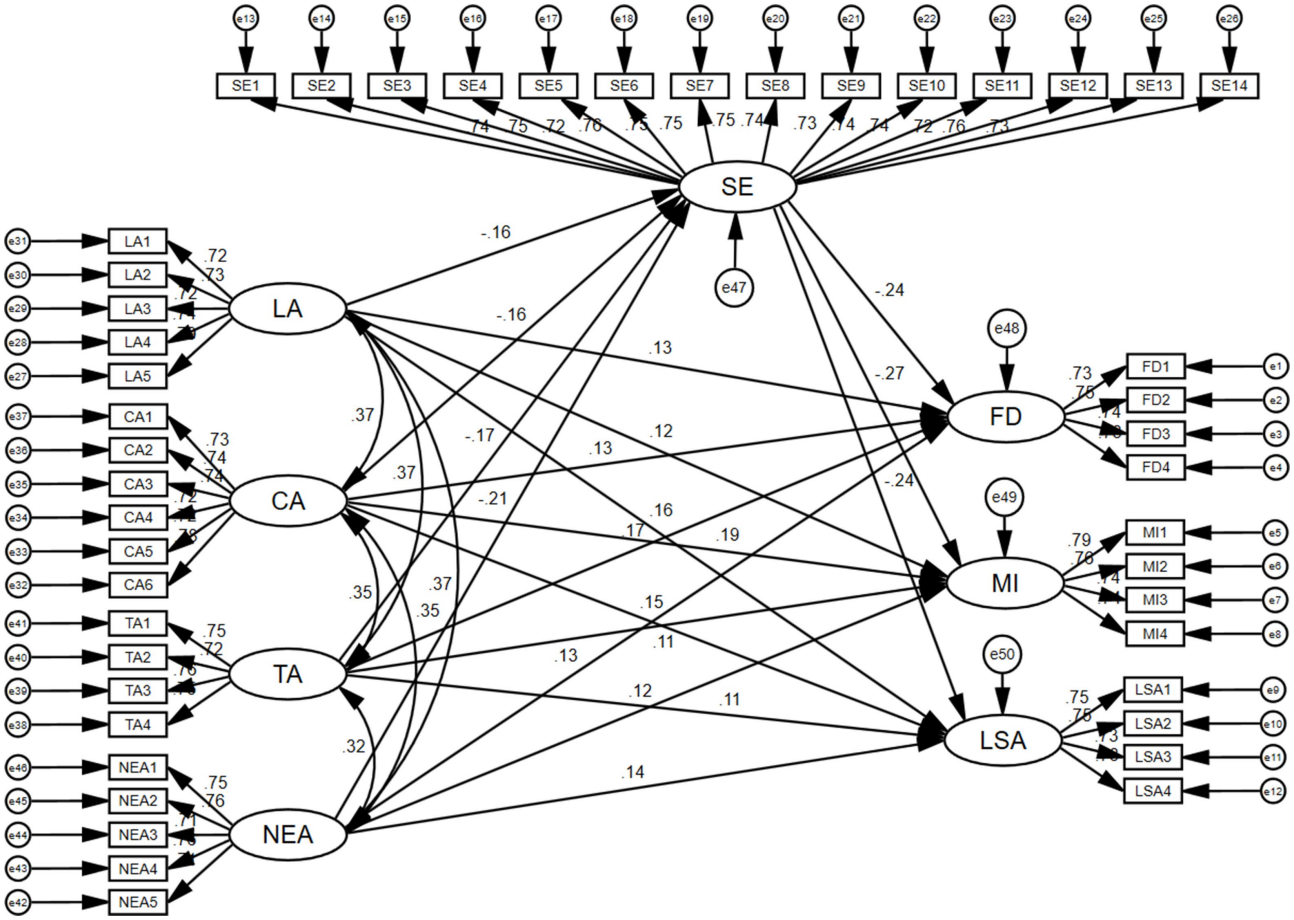
The finalized SEM model.

SEM is a statistical analysis technique used to evaluate the viability of a given theoretial model with sample data. In an effort to confirm the validity of the proposed model, each retrieved set of data was characterized utilizing goodness of fit indices and SEM (Figure 2). The SEM model exhibited satisfactory fit indices, with RMSEA and RMR values of 0.015 and 0.043, respectively, and GFI, CFI, NFI, and IFI values of 0.922, 0.990, 0.918, and 0.991, respectively.

### 4.3 Mediation effect test

The mediation effects listed in the table are statistically significant (see Table 2). Specifically, language anxiety (LA) indirectly impacts depression (FD), misconduct(MI), and a low sense of accomplishment (LSA) through academic self-efficacy (ASE), with effect sizes ranging from 0.034 to 0.043. Classroom anxiety (CA) has similar indirect effects, with effect sizes ranging from 0.034 to 0.043. Test anxiety (TA) shows slightly higher indirect effects compared to language and classroom anxiety, with effect sizes between 0.040 and 0.050. Negative evaluation anxiety (NEA) has the strongest indirect effects, with effect sizes ranging from 0.049 to 0.062.

**Table 2:** The moderated-mediating effect of LA on AB via ASE.

Path	β	Boot SE	95% CI	P
LA → ASE → FD	0.034	0.014	[0.013, 0.071]	0.001
LA → ASE → MI	0.043	0.016	[0.017, 0.082]	0.001
LA → ASE → LSA	0.036	0.014	[0.015, 0.073]	0.001
CA → ASE → FD	0.034	0.014	[0.014, 0.070]	0.001
CA → ASE → MI	0.043	0.016	[0.018, 0.085]	0.000
CA → ASE → LSA	0.035	0.014	[0.014, 0.071]	0.001
TA → ASE → FD	0.040	0.014	[0.018, 0.078]	0.000
TA → ASE → MI	0.050	0.018	[0.023, 0.094]	0.000
TA → ASE → LSA	0.041	0.015	[0.018, 0.079]	0.000
NEA → ASE → FD	0.049	0.018	[0.024, 0.096]	0.000
NEA → ASE → MI	0.062	0.021	[0.030, 0.114]	0.000
NEA → ASE → LSA	0.051	0.018	[0.025, 0.097]	0.000

This table presents that ASE mediates the relationship between anxiety (language, classroom, test, and negative evaluation) and negative psychological or behavioral outcomes. This supports that H4 holds true.

### 4.4 Independent samples T-test

The independent samples T-test was used to examine whether there were significant differences in ASE, LA and AB between students from different grades. As shown in Table 3, the results indicate that there are significant differences between undergraduate and graduate students in terms of ASE, LA and AB. Specifically, graduate students exhibited higher levels of LA and AB, while undergraduates had significantly higher ASE (M = 3.065, SD = 0.896) compared to graduate students (M = 2.513, SD = 0.757), with a T-value of 7.739 (*p* < 0.001). These findings suggest that graduate students are likely experiencing greater academic pressure and psychological stress, leading to increased anxiety and burnout, while also negatively impacting their ASE, verifying H5.

**Table 3:** Analysis of differences between undergraduate and graduate students.

	LA		ASE		AB	
	M ± SD	T	M ± SD	T	M ± SD	T
Bachelor	2.893 ± 0.568	−14.969***	3.065 ± 0.896	7.739***	2.804 ± 0.587	−17.965***
Master	3.581 ± 0.480		2.513 ± 0.757		3.673 ± 0.532	

***p < 0.001.

Results of a one-way ANOVA revealed significant differences in LA, ASE and AB among undergraduate students across grades (first-year undergraduates to fourth-year undergraduates). LA gradually increased with grade level, being lowest in freshman year (M = 2.503, SD = 0.560) and highest in senior year (M = 3.352, SD = 0.356), *F* = 52.660, *p* < 0.001. ASE, on the other hand, showed a decreasing trend, being highest in freshman year (M = 3.606, SD = 0.693) and lowest in senior year (M = 2.608, SD = 0.617), *F* = 24.800, *p* < 0.001. Regarding AB, it was lowest in freshman year (M = 2.389, SD = 0.609), and increased significantly in junior year (M = 3.155, SD = 0.300) and senior year (M = 3.184, SD = 0.384), *F* = 50.108, *p* < 0.001 (see Table 4). The results indicated that as the grade level increased, the international students increased the amount and difficulty of Chinese character learning, leading to increased LA and AB and decreased ASE, which may be closely related to the increased study load, the difficulty of Chinese character mastery, and the increasing academic pressure, reflecting the psychological challenges of international students in adapting to the linguistic and cultural environments.

**Table 4:** Analysis of differences in LA, ASE, and AB among undergraduate students.

	LA (M ± SD)	ASE (M ± SD)	AB (M ± SD)
First-year undergraduates	2.503 ± 0.560	3.606 ± 0.693	2.389 ± 0.609
Second-year undergraduates	2.744 ± 0.501	3.106 ± 1.149	2.668 ± 0.503
Third-year undergraduates	3.138 ± 0.327	2.728 ± 0.616	3.155 ± 0.300
Fourth-year undergraduates	3.352 ± 0.356	2.608 ± 0.617	3.184 ± 0.384
F	52.660***	24.800***	50.108***
LSD	1 < 2 < 3 < 4	1 > 2 > 3,4	1 < 2 < 3,4

***p < 0.001.

Postgraduate students’ LA and AB in Chinese character learning increased significantly with the grade level (see Table 5), and in particular, LA (M = 3.903, SD = 0.437) and AB (M = 4.064, SD = 0.489) were the highest in the third year of study, which were significantly higher than those in the first and second years of study. This may stem from the heavier academic pressure, study tasks, and career development anxiety faced by the third-year graduate students. In addition, ASE in the second year (M = 2.285, SD = 0.499) was significantly lower than that in the first year (M = 2.713, SD = 0.442) and the third year (M = 2.527, SD = 1.138), reflecting that students at the higher grade levels faced increased challenges in the learning mastery and use of Chinese characters, which further weakened ASE. The results suggest that as the grade level increases, graduate students are subject to greater academic and research pressures in Chinese character learning, and their motivation and self-efficacy are also affected.

**Table 5:** Differential analysis of LA, ASE and AB among graduate students.

	LA (M ± SD)	ASE (M ± SD)	AB (M ± SD)
First-year postgraduates	3.381 ± 0.371	2.713 ± 0.442	3.396 ± 0.370
Second-year postgraduates	3.506 ± 0.477	2.285 ± 0.499	3.619 ± 0.509
Third-year postgraduates	3.903 ± 0.437	2.527 ± 1.138	4.064 ± 0.489
F	31.295***	7.079**	43.213***
LSD	5,6 < 3	6 < 5,7	5 < 6 < 7

**p < 0.01 and ***p < 0.001.

### 4.5 Multivariate regression analysis

In order to explore the causes of LA, ASE and AB, logistic regression models was used to analyse the effects of personal information (gender, grade, major, region, personality, weight, parental education, and sleep quality) on these three major psychological states of learning. The model contains eight independent variables and three dependent variables, and the results of the study are shown in Table 6.

**Table 6:** Multiple regression analyses of student characteristic variables on ASE, LA and AB.

	Model 1: ASE		Model 2: LA		Model 3: AB	
	Beta	t	Beta	t	Beta	t
Grade	−0.210	−2.781**	0.274	4.931***	0.291	5.605***
Gender	0.049	1.268	0.023	0.803	0.023	0.858
Weight	0.006	0.160	−0.013	−0.476	0.011	0.425
Sleep quality	0.342	4.168***	−0.657	−10.847***	−0.576	−10.203***
Extroversion	0.214	3.163**	−0.040	−0.804	0.058	1.259
Parental education level	0.163	3.134**	0.179	4.675***	0.139	3.902***
Speciality: Liberal Arts with reference to variables	−0.007	−0.173	−0.040	−1.424	0.022	0.818
Region: Asia with reference to variables	−0.061	−1.602	−0.022	−0.786	0.004	0.162
R^2^	0.242		0.588		0.642	
F	21.035***		94.374***		118.29***	

*p < 0.05, **p < 0.01, and ***p < 0.001.

From the results of multiple regression analyses, it can be seen that grade level has a significant effect on LA, ASE and AB. The standardized coefficient of grade on ASE is −0.210 (t = −2.781, *p* < 0.01), indicating that the higher the grade, the lower the ASE. The standardized coefficient of grade level on LA was 0.274 (t = 4.931, *p* < 0.001), indicating that the level of LA increases significantly with increasing grade level. The standardized coefficient of grade on AB was 0.291 (t = 5.605, *p* < 0.001), also indicating that an increase in grade is associated with an increase in the level of AB.

The effects of gender, weight, major (with liberal arts as the reference variable), and region (with Asia as the reference variable) on ASE, LA, and AB were not significant. The standardized coefficients for gender, weight, major and region were close to zero for ASE, LA, and AB, suggesting a limited effect of gender, weight, major and region on all three.

The effect of sleep quality was significant in all three areas. The standardized coefficient of ASE was 0.342 (t = 4.168, *p* < 0.001), indicating that higher quality of sleep is associated with higher ASE. The standardized coefficient of LA was −0.657 (t = −10.847, *p* < 0.001), indicating that better quality of sleep contributes to less LA. The standardized coefficient of AB was −0.576 (t = −10.203, *p* < 0.001), indicating that higher quality of sleep was associated with lower levels of AB.

The effects of personality and parental education on all three were more complex. The standardized coefficient of personality on ASE was 0.214 (t = 3.163, *p* < 0.01), indicating that personality had a significant effect on ASE, but not on LA and AB. The standardized coefficient of parental education on ASE is 0.163 (t = 3.134, *p* < 0.01), on LA is 0.179 (t = 4.675, *p* < 0.001), and on AB is 0.139 (t = 3.902, *p* < 0.001) showing that parental education has a significant positive effect on all three variables.

## 5 Discussion

This study found that LA significantly predicts AB (*β* = 0.606, *p* < 0.001), supporting existing research on foreign language learning anxiety (Horwitz et al., 1986; Saito et al., 1999; Steel, 2010; Fernández-Castillo, 2021). The relationship operates through several key mechanisms: first, LA impairs cognitive functions like attention and memory retrieval (Moran, 2016) while increasing learning difficulties, with severe cases leading to reduced academic performance—though moderate anxiety may provide motivational benefits. The challenges are particularly pronounced in Chinese character acquisition due to the language’s unique ideographic nature, which requires mastery of approximately 3,000 characters (Wu, 2020) and presents distinct phonological/semantic processing demands (Wang et al., 2005). Our analysis revealed LA’s strongest impact was on diminishing learners’ sense of accomplishment (LSA: β = 0.145, *p* < 0.004), suggesting LA primarily undermines perceived competence—a critical pathway to AB (Schaufeli et al., 2002). Importantly, while some LA represents a normal adaptive response, prolonged exposure leads to eroded ASE and negative emotional consequences, highlighting the need to monitor and manage anxiety levels in International learners in China, with interventions focusing both on reducing excessive anxiety and preventing its chronicity to safeguard learning persistence and psychological well-being.

Evidence from mediation analysis indicates a full mediating role of ASE in the LA → AB association. This aligns with Bandura’s (1997) theory that self-efficacy buffers anxiety effects. Additionally, in line with earlier studies, this study discovered that students’ AB decreased with increasing ASE (Yang and Farn, 2005). Higher ASE people are better equipped to use constructive coping mechanisms to deal with difficulties, which is projected onto academic problems as being able to awaken learners’ motivation to learn (Liem et al., 2008). Although LA affects students’ AB and even self-efficacy, the emotional arousal function and self-efficacy will able students to suppress their LA (Dogan, 2015), with will ultimately increase the desire to learn and reduce the level of AB in Chinese characters learning. Crucially, ASE’s mediating effect was most pronounced for negative evaluation anxiety (NEA → ASE → MI: β = 0.062, *p* < 0.001), implying that learners who doubt their ability to meet external expectations are most vulnerable to burnout. This underscores ASE’s protective role in sustaining motivation amid cultural-linguistic challenges (Fan and Cui, 2024).

The independent samples T-test showed graduate learners had higher LA and AB, while undergraduates had greater ASE, due to differing academic pressures, which is in line with the study done by Chong et al. (2018). Among undergraduates, LA rose, ASE fell, and AB worsened from freshman to senior year, because of increasing academic demands. The multivariate regression analysis revealed grade level as a key predictor, with higher grades linked to lower ASE and higher LA/AB, which is consistent with previous findings (Pajares, 2003; Shen, 2005; Liu and Jackson, 2008). Good sleep quality was crucial, boosting ASE and reducing LA/AB (Pilcher et al., 1997; Okano et al., 2019; Galambos et al., 2009). Personality mainly affected self-efficacy (Chemers et al., 2001; Richardson et al., 2012), and higher parental education positively influenced all three variables (Davis-Kean, 2005; Veas et al., 2018), while gender, weight, major, and region had no significant impact.

From a theoretical perspective, the current study further confirms the control-value theory (Pekrun, 2006), which posits that the more engaged and confident conduct learners perform during the learning process, the easier it is to dissolve negative emotions, such as self-efficacy, help mitigate learners’ AB. Students with low ASE may be more prone to experiencing high levels of LA, particularly when faced with a complex task such as learning Chinese characters. If they do not feel confident in their abilities, they might interpret challenges as signs of failure, which intensifies their anxiety.

The negative correlation between ASE and AB observed in this study can be explained through the conservation of resources theory by Hobfoll (1989), which posits that individuals seek to acquire, protect, and conserve resources, such as time, energy, and self-efficacy. When learners invest significant effort in learning Chinese characters but feel that the returns (such as academic success or recognition) are insufficient, they may experience stress, anxiety and burnout. This is particularly relevant when students face language learning tasks that seem overwhelming. Besides, students may reduce their personal involvement to minimize the loss of individual resources when they perceive that the effort is not proportional to the gain during learning (Liu et al., 2023). This circumstance also explains why learning burnout is inversely proportional to the academic self-efficacy. These findings are in congruence with the investigation of Maroco et al. (2020).

In addition, the cultural and contextual factors of international students in China can further influence the interplay between ASE, LA, and AB. These students often face additional stressors such as language barriers, cultural adaptation challenges, and pressure to perform academically. These stressors can amplify feelings of anxiety, especially if students feel they are not meeting expectations or if they struggle with the complexity of learning Chinese characters. Low ASE can exacerbate these challenges, leading to a cycle of anxiety and burnout (Komarraju and Nadler, 2013).

In Chinese culture, academic success is highly valued, and learners would experience significant pressure to excel. This can undermine ASE if students feel they are not meeting these high standards, leading to increased LA and, eventually, AB. On the other hand, fostering ASE through positive reinforcement and realistic goal-setting may help students manage these pressures more effectively. These findings not only support Bandura’s social cognitive theory (Bandura, 1977) but also provide strong support for Fredrickson’s (2001, 2003) undoing effects hypothesis of positive emotions, which suggests that positive emotions mitigate the detrimental consequences of negative emotions. Specifically, Fredrickson’s work was published in 2001 in the American Psychologist (Fredrickson, 2001) and in 2003 in the American Scientist (Fredrickson, 2003). On the other hand, grounded in the control-value theory (Pekrun, 2006), it has been noted that ASE beliefs can serve as an indicator of learning-related situations and thus arouse different academic emotions.

## 6 Conclusion and implications

To conclude, the present study explored the relationship between ASE, LA and AB in Chinese characters learning among Chinese international students. The six hypotheses formulated at the beginning of the study were fully confirmed by this empirical study. As Dewaele and Li (2021) mentioned, the fundamental role of teachers in shaping student’s engagement was also significant. Based on these findings, it is recommended that educators implement the following approaches.

Firstly, it is essential to strengthen the cultivation of ASE by using AI technology to provide instant, personalized learning feedback, helping students track their progress and feel a sense of achievement (Chen and Li, 2010; Woolf et al., 2013). Teachers could introduce character-learning tasks in incremental difficulty tiers (e.g., radicals → simple characters → compounds) to reduce anxiety. Intelligent Chinese character learning apps that utilize Natural Language Processing (NLP) can adjust the difficulty of tasks based on student performance, ensuring content aligns with their actual learning level and reducing anxiety caused by overly challenging material (Yang, 2006). For immediate classroom integration, AI-powered platforms like Duolingo and Quizlet’s AI features can be deployed on student tablets to provide real-time stroke-by-stroke writing feedback through integrated digital writing pads. Secondly, to alleviate study anxiety and burnout, emotion recognition technology can monitor emotional changes during the learning process and offer timely psychological support or adjust learning strategies when signs of anxiety or burnout emerge (Vygotsky, 1978). AI can also provide instant feedback on writing errors and use big data to track learning behaviors, helping educators detect early signs of anxiety or burnout and offer targeted support. Additionally, customized support for students of different grades and individual differences is necessary (Fu, 2024); learning content should be designed to match their development stage and learning needs, while considering factors like sleep quality, personality, and parental education levels (Chen and Duh, 2009). Moreover, optimizing the learning experience with AI, through recommendation systems and interactive platforms, can enhance learning efficiency and engagement. Gamified learning and virtual writing competitions can stimulate motivation, making learning more enjoyable. Finally, it is important to strengthen home-school cooperation and psychological support by establishing communication platforms between parents and schools, providing regular updates on students’ learning and emotional states, and encouraging parental involvement (Zhang and Goodson, 2011). Psychological health services, including online counseling and educational lectures, can further assist in resolving students’ psychological challenges, thereby promoting a positive learning experience and supporting their overall well-being.

One of the main limitations of this study is its reliance on self-report measures. While the *Foreign Language Learning Anxiety Scale* (FLLAS), *Academic Burnout Scale* (ABS), and *Academic Self-Efficacy Scale* (ASES) are widely used and validated instruments, self-reported data can be prone to bias, as students may underestimate or overestimate their capabilities and feelings of anxiety due to social desirability or other factors. Another limitation is the cross-sectional design precludes causal inferences about the relationships between LA, AB, and ASE; longitudinal or experimental designs are needed to establish temporality and mechanisms. Additionally, an important limitation is the study’s failure to account for participants’ socioeconomic status (SES) or prior language learning experiences as students from higher SES backgrounds or with character-based language experience (e.g., Japanese kanji learners) may demonstrate systematically different anxiety/burnout and academic self-efficacy patterns (Lu, 2022). Future research should address these limitations by considering ways to mitigate biases in self-report data, ensuring a more balanced representation of genders, and exploring the impact of anxiety on other components of Chinese language learning to provide a more comprehensive understanding of international students’ experiences with anxiety and self-efficacy.

## Data Availability

The original contributions presented in the study are included in the article/Supplementary material, further inquiries can be directed to the corresponding author.

## References

[ref1] Bandura A. (1977). Self-efficacy: toward a unifying theory of behavioral change. Psychol. Rev. 84, 191–215. doi: 10.1037/0033-295X.84.2.191, PMID: 847061

[ref2] Bandura A. (1986). Social foundations of thought and action: a social-cognitive view. Acad. Manag. Rev. 11, 803–820. doi: 10.2307/258004

[ref3] Bandura A. (1997). Self-efficacy: The exercise of control. New York, NY: W.H. Freeman and Company.

[ref4] Bassetti B. Mairano P. Masterson J. Cerni T. (2020). Effects of orthographic forms on second language speech production and phonological awareness, with consideration of speaker-level predictors. Lang. Learn. 70, 1218–1256. doi: 10.1111/lang.12423, PMID: 40421581

[ref5] Britner S. L. Pajares F. (2006). Sources of science self-efficacy beliefs of middle school students. J. Res. Sci. Teach. 43, 485–499. doi: 10.1002/tea.20131

[ref6] Caprara G. V. Vecchione M. Alessandri G. Gerbino M. Barbaranelli C. (2011). The contribution of personality traits and self-efficacy beliefs to academic achievement: a longitudinal study. Br. J. Educ. Psychol. 81, 78–96. doi: 10.1348/2044-8279.002004, PMID: 21199485

[ref1000] Chai X. Bao J. (2023). Linguistic distances between native languages and Chinese influence acquisition of Chinese character, vocabulary, and grammar. Frontiers in Psychology 13. doi: 10.3389/fpsyg.2022.1083574 PMC9880333 36710744

[ref8] Charkhabi M. Abarghuei M. A. Hayati D. (2013). The association of academic burnout with self-efficacy and quality of learning experience among Iranian students. Springerplus 2:677. doi: 10.1186/2193-1801-2-677 24386623 PMC3872283

[ref9] Chemers M. M. Hu L. Garcia B. F. (2001). Academic self-efficacy and first-year college student performance and adjustment. J. Educ. Psychol. 93, 55–64. doi: 10.1037/0022-0663.93.1.55

[ref10] Chen C. M. Duh L. J. (2009). Personalized web-based tutoring system based on fuzzy item response theory. Expert Syst. Appl. 36, 8739–8753. doi: 10.1016/j.eswa.2007.03.010

[ref11] Chen C. M. Li Y. L. (2010). Personalized context-aware ubiquitous learning system for supporting effective English vocabulary learning. Interact. Learn. Environ. 18, 341–364. doi: 10.1080/10494820802602329

[ref12] Cheng Y.-H. Tsai C.-C. Liang J.-C. (2019). Academic hardiness and academic self-efficacy in graduate studies. High. Educ. Res. Dev. 38, 907–921. doi: 10.1080/07294360.2019.1612858

[ref13] Chong W. H. Liem G. A. D. Huan V. S. Kit P. L. Ang R. P. (2018). Student perceptions of self-efficacy and teacher support for learning in fostering youth competencies: roles of affective and cognitive engagement. J. Adolesc. 68, 1–11. doi: 10.1016/j.adolescence.2018.07.002, PMID: 29986166

[ref14] Davis-Kean P. E. (2005). The influence of parent education and family income on child achievement: the indirect role of parental expectations and the home environment. J. Fam. Psychol. 19, 294–304. doi: 10.1037/0893-3200.19.2.294, PMID: 15982107

[ref16] Dewaele J.-M. Chen X. Padilla A. M. Lake J. (2019). The flowering of positive psychology in foreign language teaching and acquisition research. Front. Psychol. 10:2128. doi: 10.3389/fpsyg.2019.02128, PMID: 31607981 PMC6769100

[ref17] Dewaele J.-M. Leung P. P. Y. (2022). The effect of proficiency on non-native English as a foreign language (EFL) teachers' self-efficacy and practice in the EFL classroom. IAFOR J. Educ. 10, 11–32. doi: 10.22492/ije.10.1.01

[ref18] Dewaele J.-M. Li C. (2021). Teacher enthusiasm and students' social-behavioral learning engagement: the mediating role of student enjoyment and boredom in Chinese EFL classes. Lang. Teach. Res. 25, 922–945. doi: 10.1177/13621688211014538, PMID: 40441278

[ref19] Dewaele J.-M. MacIntyre P. D. (2014). The two faces of Janus? Anxiety and enjoyment in the foreign language classroom. Stud. Second Lang. Learn. Teach. 4, 237–274. doi: 10.14746/ssllt.2014.4.2.5

[ref1001] Dogan U. (2015). Student engagement, academic self-efficacy, and academic motivation as predictors of academic performance. The Anthropologist 20, 553–561. doi: 10.1080/09720073.2015.11891759

[ref21] Dörnyei Z. (2005). The psychology of the language learner: Individual differences in second language acquisition. Mahwah, NJ: Lawrence Erlbaum Associates.

[ref1002] Fan L. Cui F. (2024). Mindfulness, self-efficacy, and self-regulation as predictors of psychological well-being in EFL learners. Frontiers in Psychology 15, 1332002. doi: 10.3389/fpsyg.2024.1332002 38601825 PMC11004504

[ref25] Fan Z. Hu J. (2010). Cultural conflicts and adaptation of international students from Central-Asia. J. Xinjiang Normal Univ. (Philos. Soc. Sci. Ed.). 31, 107–114. doi: 10.14100/j.cnki.65-1039/g4.2010.03.011

[ref26] Fernández-Castillo A. (2021). State-anxiety and academic burnout regarding university access selective examinations in Spain during and after the COVID-19 lockdown. Front. Psychol. 12:621863. doi: 10.3389/fpsyg.2021.621863, PMID: 33584481 PMC7873299

[ref28] Fredrickson B. L. (2001). The role of positive emotions in positive psychology: the broaden-and-build theory of positive emotions. Am. Psychol. 56, 218–226. doi: 10.1037/0003-066X.56.3.218, PMID: 11315248 PMC3122271

[ref29] Fredrickson B. L. (2003). The value of positive emotions: The emerging science of positive psychology is coming to understand why it’s good to feel good. American Scientist 91, 330–335.

[ref30] Freudenberger H. J. (1974). Staff burn-out. J. Soc. Issues 30, 159–165. doi: 10.1111/j.1540-4560.1974.tb00706.x

[ref31] Fu L. (2024). Social support in class and learning burnout among Chinese EFL learners in higher education: are academic buoyancy and class level important? Curr. Psychol. 43, 5789–5803. doi: 10.1007/s12144-023-04778-9, PMID: 37359569 PMC10215065

[ref32] Galambos N. L. Dalton A. L. Maggs J. L. (2009). Losing sleep over it: daily variation in sleep quantity and quality in Canadian students' first semester of university. J. Res. Adolesc. 19, 741–761. doi: 10.1111/j.1532-7795.2009.00618.x

[ref33] Han Y. Wang Y. (2021). Investigating the correlation among Chinese EFL teachers’ self-efficacy, work engagement, and reflection. Front. Psychol. 12:763234. doi: 10.3389/fpsyg.2021.763234, PMID: 34803845 PMC8603392

[ref34] Hobfoll S. E. (1989). Conservation of resources: a new attempt at conceptualizing stress. Am. Psychol. 44, 513–524. doi: 10.1037/0003-066X.44.3.513, PMID: 2648906

[ref35] Hobfoll S. E. Halbesleben J. Neveu J.-P. Westman M. (2018). Conservation of resources in the organizational context: the reality of resources and their consequences. Annu. Rev. Organ. Psych. Organ. Behav. 5, 103–128. doi: 10.1146/annurev-orgpsych-032117-104640

[ref36] Honicke T. Broadbent J. (2016). The influence of academic self-efficacy on academic performance: a systematic review. Educ. Res. Rev. 17, 63–84. doi: 10.1016/j.edurev.2015.11.002

[ref37] Horwitz E. K. Horwitz M. B. Cope J. (1986). Foreign language classroom anxiety. Mod. Lang. J. 70, 125–132. doi: 10.1111/j.1540-4781.1986.tb05256.x

[ref38] Kan Y. Wan B. Chen Y. Qiu X. Qiao Z. Zhou J.. (2025). Longitudinal correlates of learning burnout among Chinese adolescents during the COVID-19 pandemic: a cross-lagged panel network analysis. J. Affect. Disord. 369, 125–134. doi: 10.1016/j.jad.2024.09.137 39321978

[ref39] Klassen R. M. Usher E. L. (2010). “Self-efficacy in educational settings: recent research and emerging directions” in The decade ahead: Theoretical perspectives on motivation and achievement. eds. Urdan T. C. Karabenick S. A. (United Kingdom: Emerald), 1–33.

[ref40] Komarraju M. Nadler D. (2013). Self-efficacy and academic achievement: why do implicit beliefs, goals, and effort regulation matter? Learn. Individ. Differ. 25, 67–72. doi: 10.1016/j.lindif.2013.01.005

[ref41] Krashen S. (1985). The input hypothesis: Issues and implications. London: Longman.

[ref42] Lee S. H. Jeon W. T. (2015). The relationship between academic self-efficacy and academic burnout in medical students. Korean J. Med. Educ. 27, 27–35. doi: 10.3946/kjme.2015.27.1.27, PMID: 25800259 PMC8813535

[ref43] Lepp A. Barkley J. E. Karpinski A. C. (2014). The relationship between cell phone use, academic performance, anxiety, and satisfaction with life in college students. Comput. Hum. Behav. 31, 343–350. doi: 10.1016/j.chb.2013.10.049

[ref44] Li S. (2015). The associations between language aptitude and second language grammar acquisition: a meta-analytic review of five decades of research. Appl. Linguis. 36, 385–408. doi: 10.1093/applin/amu054

[ref45] Li Jingguo. (2019). Research on Teaching Chinese Characters to Foreigners from the Perspective of Chinese Character Culture (Master's thesis, Shaanxi Normal University). Master's degree. https://kns.cnki.net/kcms2/article/abstract?v=qQX4xeHgc6utXstqW7Em-WRVUwraXs8CC7-v6DIqFxWUJlsEtCZZPiODLjow9nZAaA8pLqXw7dZCmoWr-rJ774ssGw_yJeYf_uv2qfi3Xgh2mM4psVh_tQ3A8x6_xd_SIez9vIx9kfLHhoH1DkWsUT-Raak3oUeqAft8gIQZ7jbZelTFMXv8_1MYZcJOV355QNfPDjxYbRI=&uniplatform=NZKPT&language=CHS

[ref46] Li C. Zhang L. J. Jiang G. (2021). Conceptualisation and measurement of foreign language learning burnout among Chinese EFL students. J. Multiling. Multicult. Dev. 42, 906–920. doi: 10.1080/01434632.2021.1931246

[ref47] Liem A. D. Lau S. Nie Y. (2008). The role of self-efficacy, task value, and achievement goals in predicting learning strategies, task disengagement, peer relationships, and achievement outcome. Contemp. Educ. Psychol. 33, 486–512. doi: 10.1016/j.cedpsych.2007.08.001

[ref48] Liu M. (2006). Anxiety in Chinese EFL students at different proficiency levels. System 34, 301–316. doi: 10.1016/j.system.2006.04.004

[ref49] Liu L. (2010). *A study of Chinese reading anxiety among international students in Central Asia [Master’s thesis, Xinjiang Normal University]*: CNKI, from https://kns.cnki.net/kcms2/article/abstract?v=b41E60TuiN_B0M8irgz4FDlBy0Ppucsm_xG7X_9bpQJreBpjCxvoFgIjCheJFHAcPTneJ3faAp6dSXH7ZTSpd1DJE28IEobcwTDFGnhErTWJa8HOcpKHMfEq0VS_s79zFgT9V6Sr5UhuZ-7kIV3EYX1akv3_vKAt6pTGl1N0bBG4qv-CyoyIcK-3SfMDzb0e&uniplatform=NZKPT&language=CHS (Accessed January 16, 2012).

[ref50] Liu J. J.. (2023). A survey study on reading anxiety among intermediate and advanced level Chinese learners in Russian Confucius Institutes (Master’s thesis, Dalian University of Foreign Languages). CNKI. doi: 10.26993/d.cnki.gslyc.2023.000391

[ref51] Liu Y. Hsieh P. (2016). The impact of cognitive load on learning Chinese characters among foreign learners. Educ. Stud. 42, 203–220. doi: 10.1080/03055698.2015.1117312

[ref52] Liu M. Jackson J. (2008). An exploration of Chinese EFL learners' unwillingness to communicate and foreign language anxiety. Mod. Lang. J. 92, 71–86. doi: 10.1111/j.1540-4781.2008.00687.x

[ref53] Liu H. Zhong Y. Chen H. Wang Y. (2023). The mediating roles of resilience and motivation in the relationship between students' English learning burnout and engagement: a conservation-of-resources perspective. Int. Rev. Appl. Linguist. Lang. Teach. 63:89. doi: 10.1515/iral-2023-0089

[ref97] Lu Xiaoming. (2022). Research on the construction of translation competence model for Japanese undergraduates and its application in translation teaching (Doctoral dissertation, Fujian Normal University). Ph.D. CNKI. doi: 10.27019/d.cnki.gfjsu.2022.002159

[ref54] Luo T.-Y. Qian D. Bellassen J. (2025). From the poetic nature of Chinese characters to the “character-based” pedagogy: an interview with French sinologist prof Le sang Bailly. Int. Sinol. 2, 130–137. doi: 10.19326/j.cnki.2095-9257.2025.02.015

[ref55] Lyndon M. P. Henning M. A. Alyami H. Krishna S. Zeng I. Yu T. C.. (2017). Burnout, quality of life, motivation, and academic achievement among medical students: A person-oriented approach. Perspectives on medical education 6, 108–114. doi: 10.1007/s40037-017-0340-6 28247209 PMC5383573

[ref56] MacIntyre P. D. Gregersen T. (2012). Emotions that facilitate language learning: the positive-broadening power of the imagination. Stud. Second Lang. Learn. Teach. 2:193. doi: 10.14746/ssllt.2012.2.2.4

[ref57] Maroco J. Assunção H. Harju-Luukkainen H. Lin S.-W. Sit P.-S. Cheung K.. (2020). Predictors of academic efficacy and dropout intention in university students: can engagement suppress burnout? PLoS One 15:e0239816. doi: 10.1371/journal.pone.0239816, PMID: 33119598 PMC7595383

[ref58] Martin A. J. Rimm-Kaufman S. E. (2015). Do student self-efficacy and teacher-student interaction quality contribute to emotional and behavioral engagement in fifth grade math? J. Sch. Psychol. 53, 359–373. doi: 10.1016/j.jsp.2015.07.001, PMID: 26407834

[ref60] May R. W. Bauer K. N. Fincham F. D. (2015). School burnout: diminished academic and cognitive performance. Learn. Individ. Differ. 42, 126–131. doi: 10.1016/j.lindif.2015.07.015

[ref62] Mills N. Pajares F. Herron C. (2007). Self-efficacy of college intermediate French students: relation to achievement and motivation. Lang. Learn. 57, 417–442. doi: 10.1111/j.1467-9922.2007.00421.x

[ref63] Mokhtar M. M. Abidin M. J. Z. (2021). Well-being and academic workload: perceptions of science and technology students. Educ. Res. Rev. 16, 418–427. doi: 10.5897/ERR2021.4197

[ref64] Moran T. P. (2016). Anxiety and working memory capacity: a meta-analysis and narrative review. Psychol. Bull. 142, 831–864. doi: 10.1037/bul0000051, PMID: 26963369

[ref65] Mulyadi S. Rahardjo W. Basuki A. M. H. (2016). The role of parent-child relationship, self-esteem, academic self-efficacy to academic stress. Procedia Soc. Behav. Sci. 217, 603–608. doi: 10.1016/j.sbspro.2016.02.063

[ref66] Okano K. Kaczmarzyk J. R. Dave N. Gabrieli J. D. E. Grossman J. C. (2019). Sleep quality, duration, and consistency are associated with better academic performance in college students. NPJ Sci. Learn. 4:16. doi: 10.1038/s41539-019-0055-z 31583118 PMC6773696

[ref67] Orakcı Ş. Yüregilli Göksu D. Karagöz S. (2023). A mixed methods study of the teachers’ self-efficacy views and their ability to improve self-efficacy beliefs during teaching. Front. Psychol. 13:1035829. doi: 10.3389/fpsyg.2022.1035829, PMID: 36687810 PMC9845933

[ref68] Pajares F. (1996). Self-efficacy beliefs in academic settings. Rev. Educ. Res. 66, 543–578. doi: 10.3102/00346543066004543

[ref69] Pajares F. (2003). Self-efficacy beliefs, motivation, and achievement in writing: a review of the literature. Read. Writ. Q. 19, 139–158. doi: 10.1080/10573560308222

[ref71] Pekrun R. (2006). The control-value theory of achievement emotions: assumptions, corollaries, and implications for educational research and practice. Educ. Psychol. Rev. 18, 315–341. doi: 10.1007/s10648-006-9029-9

[ref73] Pilcher J. J. Ginter D. R. Sadowsky B. (1997). Sleep quality versus sleep quantity: relationships between sleep and measures of health, well-being and sleepiness in college students. J. Psychosom. Res. 42, 583–596. doi: 10.1016/S0022-3999(97)00004-4, PMID: 9226606

[ref74] Richardson M. Abraham C. Bond R. (2012). Psychological correlates of university students' academic performance: a systematic review and meta-analysis. Psychol. Bull. 138, 353–387. doi: 10.1037/a0026838, PMID: 22352812

[ref75] Saito Y. Horwitz E. K. Garza T. J. (1999). Foreign language reading anxiety. Mod. Lang. J. 83, 202–218. doi: 10.1111/0026-7902.00016

[ref76] Schaufeli W. B. Martínez I. M. Pinto A. M. Salanova M. Bakker A. B. (2002). Burnout and engagement in university students: a cross-national study. J. Cross-Cult. Psychol. 33, 464–481. doi: 10.1177/0022022102033005003

[ref77] Schaufeli W. B. Taris T. W. (2005). The conceptualization and measurement of burnout: common ground and worlds apart. Work Stress. 19, 256–262. doi: 10.1080/02678370500385913

[ref78] Schunk D. H. DiBenedetto M. K. (2020). Motivation and social cognitive theory. Contemp. Educ. Psychol. 60:1832. doi: 10.1016/j.cedpsych.2019.101832

[ref79] Schwarzer R. Aristi B. (1997). Optimistic self-beliefs: assessment of general perceived self-efficacy in thirteen cultures. World Psychol. 3, 177–190.

[ref80] Schwarzer R. Bäßler J. Kwiatek P. Schröder K. Zhang J. X. (1997). The assessment of optimistic self-beliefs: comparison of the German, Spanish, and Chinese versions of the general self-efficacy scale. Appl. Psychol. 46, 69–88. doi: 10.1111/j.1464-0597.1997.tb01096.x, PMID: 40421581

[ref81] Shen H. H. (2005). An investigation of Chinese-character learning strategies among non-native speakers of Chinese. System 33, 49–68. doi: 10.1016/j.system.2004.11.001

[ref82] Shen H. H. (2013). Chinese L2 literacy development: cognitive characteristics, learning strategies, and pedagogical interventions. Lang Ling Compass 7, 371–387. doi: 10.1111/lnc3.12034

[ref83] Spielberger C. D. (1972). “Anxiety as an emotional state” in Anxiety: Current trends in theory and research. ed. Spielberger C. D., vol. 1 (New York: Academic Press), 23–49.

[ref84] Steel P. (2010). The procrastination equation: How to stop putting things off and start getting stuff done. Toronto, Canada: Random House Canada.

[ref85] Sun Y. (2025). A study on the Chinese language proficiency goals of high-level learners from south-south cooperation countries in China. Lang. Strat. Res. 10, 88–96. doi: 10.19689/j.cnki.cn10-1361/h.20250308

[ref86] Taylor I. Taylor M. M. (1995). Writing and literacy in Chinese, Korean, and Japanese. Amsterdam, Netherlands: John Benjamins Publishing.

[ref87] Tran T. T. T. Baldauf R. B. Jr. Moni K. (2012). Foreign language anxiety: understanding its status and insiders' awareness and attitudes. TESOL Q. 46, 783–803. doi: 10.1002/tesq.85

[ref88] Tweed R. G. Lehman D. R. (2002). Learning considered within a cultural context: Confucian and Socratic approaches. Am. Psychol. 57, 89–99. doi: 10.1037/0003-066X.57.2.89, PMID: 11899565

[ref89] Veas A. Castejón J.-L. Miñano P. Gilar-Corbí R. (2018). Relationship between parent involvement and academic achievement through metacognitive strategies: a multiple multilevel mediation analysis. Br. J. Educ. Psychol. 89, 393–411. doi: 10.1111/bjep.12245 30198550

[ref90] Vygotsky L. S. (1978). Mind in society: The development of higher psychological processes. Cambridge, MA: Harvard University Press.

[ref91] Wang Y. Pan Z. (2023). Modeling the effect of Chinese EFL teachers’ self-efficacy and resilience on their work engagement: a structural equation modeling analysis. SAGE Open 13:4329. doi: 10.1177/21582440231214329

[ref92] Wang M. Perfetti C. A. Liu Y. (2005). Chinese-English biliteracy acquisition: cross-language and writing system transfer. Cognition 97, 67–88. doi: 10.1016/j.cognition.2004.10.001, PMID: 16139587

[ref94] Woolf B. P. Lane H. C. Chaudhri V. K. Kolodner J. L. (2013). AI grand challenges for education. AI Magazine 34, 66–84. doi: 10.1609/aimag.v34i4.2490

[ref95] Wu H. (2020). The interplay of learning burnout and anxiety: an examination of Chinese university students in a foreign language context. Educ. Stud. Lang. Learn. 28, 350–367. doi: 10.1080/1088476X.2020.1692369

[ref99] Xu Y. Jin L. Deifell E. Angus K. (2021). Facilitating technology-based character learning in emergency remote teaching. Foreign Lang. Ann. 54, 343–370. doi: 10.1111/flan.12541

[ref100] Yang S. J. H. (2006). Context aware ubiquitous learning environments for peer-to-peer collaborative learning. Educ. Technol. Soc. 9, 188–201.

[ref101] Yang J. (2021). The predictive role of Chinese EFL teachers’ individual self-efficacy and collective efficacy in their work engagement. Front. Psychol. 12:752041. doi: 10.3389/fpsyg.2021.752041, PMID: 34603168 PMC8481583

[ref102] Yang H. J. Farn C. K. (2005). An investigation of the factors affecting MIS student burnout in technical-vocational colleges. Comput. Hum. Behav. 21, 917–932. doi: 10.1016/j.chb.2004.03.001

[ref103] Zhang Y. Gan Y. Cham H. (2007). Perfectionism, academic burnout, and engagement among Chinese college students: a structural equation modeling analysis. Pers. Individ. Differ. 43, 1529–1540. doi: 10.1016/j.paid.2007.04.010

[ref104] Zhang J. Goodson P. (2011). Predictors of international students' psychosocial adjustment to life in the United States: a systematic review. Int. J. Intercult. Relat. 35, 139–162. doi: 10.1016/j.ijintrel.2010.11.011

[ref105] Zhang J. X. Schwarzer R. (1995). Measuring optimistic self-beliefs: a Chinese adaptation of the general self-efficacy scale. Psychologia 38, 174–181.

[ref106] Zhang S. Zhu Z. Y. (2025). Research on international Chinese character teaching under multimodal teaching theory. Chin. Character Cult. 12, 99–101. doi: 10.14014/j.cnki.cn11-2597/g2.2025.06.065

[ref107] Zhao Y. (2019). Establishment and application of junior middle school students’ learning weariness scale. J. Shanghai Educ. Res. 10, 27–30. doi: 10.16194/j.cnki.31-1059/g4.2019.10.006

[ref108] Zimmerman B. J. (2000). Self-efficacy: an essential motive to learn. Contemp. Educ. Psychol. 25, 82–91. doi: 10.1006/ceps.1999.1016, PMID: 10620383

[ref109] Zimmerman B. J. Schunk D. H. (2011). Self-regulated learning and academic achievement: Theoretical perspectives. New York, NY: Routledge.

